# Progressive Unilateral Proptosis and [ ^18^ F]FDG-Avid Lesions Connoting Aggressive Disease in Radioiodine-Refractory Differentiated Thyroid Carcinoma

**DOI:** 10.1055/s-0044-1792029

**Published:** 2024-10-25

**Authors:** Yeshwanth Edamadaka, Rahul V. Parghane, Sandip Basu

**Affiliations:** 1 Radiation Medicine Centre, Bhabha Atomic Research Centre, Tata Memorial Hospital Annexe, Mumbai, Maharashtra, India; 2 Homi Bhabha National Institute, Mumbai, Maharashtra, India

**Keywords:** radioiodine-refractory differentiated thyroid carcinoma (RAIR-DTC), radioactive iodine (RAI) therapy, [18F]FDG-PET/CT scan, orbital metastasis, targeted therapy

## Abstract

Radioiodine-refractory differentiated thyroid carcinoma (RAIR-DTC) have poor prognosis as compared with radioiodine concentrating thyroid carcinoma. We present a case of follicular thyroid carcinoma presenting as a disseminated disease initially, underwent thyroid surgery and radioiodine (RAI) therapy. Following RAI, the patient developed a sudden-onset unilateral proptosis, finally diagnosed as orbital metastasis. [ ^18^ F]-Fluorodeoxyglucose (FDG)-positron emission tomography-computed tomography identified FDG-avid metastatic lesions more numerous than the RAI-avid lesions. The patient was treated with local and systemic targeted therapy, but despite these treatments, he developed progressive disease indicating aggressive nature of the disease consistent with [ ^18^ F]FDG-avid RAIR-DTC.

## Introduction

Differentiated thyroid carcinoma (DTC) is the most common type of endocrine malignant tumor. The standard of care is surgery followed by radioactive iodine (RAI) therapy in most cases of DTC. Recently, the American Thyroid Association guideline introduced the term radioiodine-refractory DTC (RAIR-DTC), which have poor prognosis with limited therapeutic options. 1 We present a patient of follicular thyroid carcinoma diagnosed from lumbar vertebral metastasis, presenting as disseminated disease initially, and treated with surgery and RAI therapy. Following RAI, he developed sudden-onset unilateral proptosis finally diagnosed as orbital metastasis from DTC.

## Case History

**Fig. 1:**
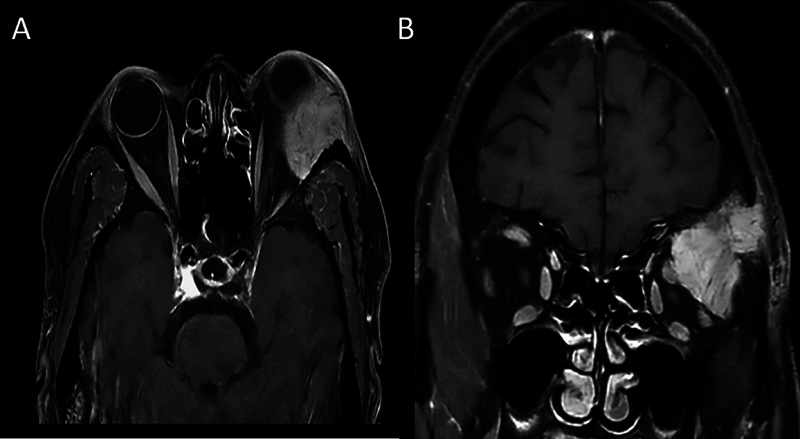
Clinical patient image showing unilateral proptosis in the left eye with epiphora ( **A** ). Axial T1 postcontrast magnetic resonance imaging (MRI) showing an ill-defined lobulated lesion in the lateral aspect of the orbit in extraconal space displacing the globe anteriorly ( **B** ). Coronal T1 postcontrast MRI showing the lesion abutting the superior and lateral rectus muscles with temporal bone invasion ( **C** ).

A 57-year-old male patient presented with chronic lower back pain which on magnetic resonance imaging (MRI) evaluation revealed multiple lytic skeletal lesions in the spine with L3 vertebral collapse. Histopathological analysis from L3 lesion showed metastatic disease from follicular thyroid carcinoma with immunohistochemistry positive for TTF-1, thyroglobulin, PAX8, and K _i_ -67 index of 15%. The patient underwent total thyroidectomy followed by high-dose RAI therapy. Post-RAI follow-up (8 months after RAI), he developed a sudden-onset swelling in the left orbital region ( Fig. 1A ). He was evaluated with MRI which revealed an ill-defined lobulated lesion in the lateral aspect of the orbit abutting the superior and lateral rectus muscles with temporal bone with invasion and no intracranial extension noted ( Fig. 1B and C ).

**Fig. 2:**
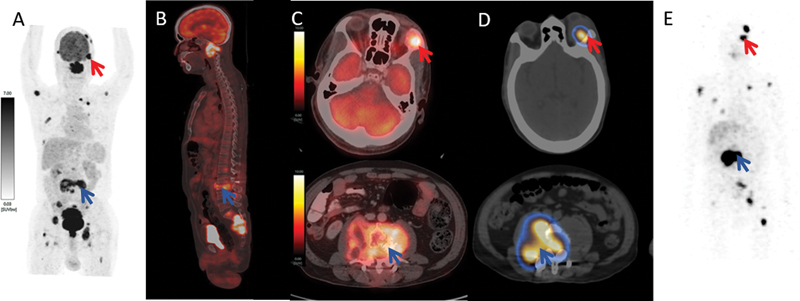
[18F]-Fluorodeoxyglucose (FDG)-positron emission tomography-computed tomography (PET/CT), maximum intensity projection (MIP) image ( **A** ) and sagittal fused image ( **B** ) demonstrating hypermetabolic multiple lytic skeletal lesions at the calvarium, left orbit (red arrow), C2 vertebra, multiple ribs, L3 vertebra (blue arrow), multiple pelvic bone, and sacrum with intraspinal extension (blue arrow). Axial fused images ( **C** ) showing [18F]FDG-avid (maximum standardized uptake value [SUVmax] 11.50) left lateral extraconal orbital metastasis (red arrow) and [18F]FDG-avid (SUVmax 14.50) L3 vertebral metastasis with compression fracture and associated soft tissue component (blue arrow). ^131^ I fused axial single-photon emission computed tomography (SPECT)/computed tomography (CT) images ( **D** ) and whole-body MIP SPECT/CT image ( **E** ) showing ^131^ I uptake in left lateral extraconal orbital metastasis, L3 vertebral lesion, and other skeletal lesions, but on comparison with [18F]FDG-PET/CT scan, ^131^ I scan showed lack of concentration in cervical and sacral metastatic lesions suggestive of dedifferentiation and aggressive disease.

He underwent [18F]-fluorodeoxyglucose (FDG)-positron emission tomography-computed tomography (PET/CT), which showed increased uptake in left orbital lesion, multiple skeletal lesions, and lung lesions ( Fig. 2A–C ). Whole-body (WB) radioiodine ( ^131^ I) single-photon emission CT/ CT, showed radioiodine uptake in the metastatic left orbital lesion and multiple skeletal lesions ( Fig. 2D and E ). The patient received a palliative external beam radiotherapy (EBRT) to the orbit lesion and lumbar vertebral lesions and was given tablet lenvatinib 14 mg once daily. He tolerated the treatment well initially, but presented with spastic paresis 3 months after EBRT and developed symptoms of weight loss and increase in orbital swelling with declining performance, which was treated as supportive treatment.

## Discussion

The recent consensus statement by the American Head and Neck Society, Endocrine Surgery Section, and International Thyroid Oncology Group has defined advanced thyroid carcinoma according to four categories including surgical, biochemical, molecular, and at discretion of clinician when features portend aggressive tumor behavior. In the literature, various studies indicated that patients of RAIR-DTC had the worst outcome with a median 10-year survival rate of 10%. 2 Before considering systemic treatments in patients with advanced thyroid carcinoma, local therapies are typically offered to symptomatic disease. The targeted therapy in RAIR-DTC is recommended in cases with a rapidly progressive tumor, symptomatic disease, and tumors in a threatening location. 3 The multikinase inhibitors for advanced thyroid carcinoma include sorafenib and lenvatinib as initial targeted therapy, while cabozantinib received approval as second-line therapy without any biomarker selection. 4 The use of targeted therapy in RAIR-DTC should be made by a multidisciplinary team while weighing risks versus benefits and close surveillance for any treatment-related side effects and disease progression. 5 Thyroid carcinoma rarely metastasizes to orbit with only 3% from thyroid origin. 6 They often present with symptoms of diplopia, ocular pain, and vision loss coupled with globe displacement and palpable masses. 7 [ ^18^ F]FDG-PET/CT is of considerable value in characterizing patients with advanced thyroid carcinoma presenting with suspected orbital metastases and further evaluating global WB disease burden. 8 For the treatment of symptomatic orbital metastasis in DTC, local EBRT is useful with overall response rates between 75 and 100%, a median local control rate of 11 months after EBRT. 9 We herein presented a rare case of progressive orbital metastasis in RAIR-DTC, demonstrating more [18F]FDG-avid lesions than radioiodine-avid disease and received local therapy as well as systemic targeted therapy, but eventually the patient developed progressive disease within a short period of time connoting aggressive nature of disease in RAIR-DTC.

## Conclusion

[18F]FDG-PET/CT plays a crucial role in identifying dedifferentiated lesions, evaluating global metastatic burden, and as a prognostic biomarker in RAIR-DTC. FDG-avidity in RAIR-DTC can signify a poor prognosis and outcome despite local therapy and systemic targeted therapy. Unilateral proptosis in known case of thyroid carcinoma should raise a suspicion of metastasis and need urgent investigation for diagnosing this rare site metastasis in DTC.
